# The Dispensable Roles of X-Linked *Ubl4a* and Its Autosomal Counterpart *Ubl4b* in Spermatogenesis Represent a New Evolutionary Type of X-Derived Retrogenes

**DOI:** 10.3389/fgene.2021.689902

**Published:** 2021-06-25

**Authors:** Changping Yu, Runjie Diao, Ranjha Khan, Cheng Deng, Hui Ma, Zhijie Chang, Xiaohua Jiang, Qinghua Shi

**Affiliations:** ^1^First Affiliated Hospital of USTC, Hefei National Laboratory for Physical Sciences at Microscale, School of Basic Medical Sciences, Division of Life Sciences and Medicine, University of Science and Technology of China, Hefei, China; ^2^Jiangsu Key Laboratory for Biodiversity and Biotechnology, College of Life Sciences, Nanjing Normal University, Nanjing, China; ^3^State Key Laboratory of Membrane Biology, National Engineering Laboratory for Anti-tumor Therapeutics, School of Medicine, Tsinghua University, Beijing, China

**Keywords:** X chromosome, retrogene, evolution, *Ubl4a*, *Ubl4b*, spermatogenesis

## Abstract

X-derived retrogenes contribute to genetic diversity in evolution and are usually specifically expressed in testis and perform important functions during spermatogenesis. *Ubl4b* is an autosomal retrogene with testis-specific expression derived from *Ubl4a*, an X-linked housekeeping gene. In the current study, we performed phylogenetic analysis and revealed that *Ubl4a* and *Ubl4b* are subject to purifying selection and may have conserved functions in evolution. *Ubl4b* was knocked out in mice using CRISPR/Cas9 genome editing technology and interestingly, we found no alterations in reproductive parameters of *Ubl4b^–/–^* male mice. To get insights into whether *Ubl4a* could compensate the absence of *Ubl4b in vivo*, we further obtained *Ubl4a^–/Y^; Ubl4b^–/–^* mice that lack both *Ubl4a* and *Ubl4b*, and the double knockout (dKO) mice also displayed normal spermatogenesis, showing that *Ubl4a* and *Ubl4b* are both dispensable for spermatogenesis. Thus, through the *in vivo* study of UBL4A and UBL4B, we provided a direct evidence for the first time that some X chromosome-derived autosomal retrogenes can be unfunctional in spermatogenesis, which represents an additional evolutionary type of X-derived retrogenes.

## Introduction

Biological diversity depends upon the possible emergence of duplicate genes in the genome of organisms, and is also related with the changes in gene regulatory pathways (Gu et al., 2004; Johnson et al., 2007; ENCODE Project Consortium, 2012; Stunnenberg et al., 2016; Roundtree et al., 2017). Among the several mechanisms of procreating new genes, retroposition has been considered as one of the important mechanisms in diverse species (Long et al., 2003; Wang et al., 2006; Bai et al., 2007). Retroposition refers to the process where reverse transcriptase converts mRNAs of progenitor genes into DNAs followed by integration into the genome to generate retrogenes (Schrader and Schmitz, 2019). Retrogenes show diverse movement patterns because of their different genomic positions compared with progenitor genes, which contributes to genetic diversity in evolution (Dai et al., 2006; Schrader and Schmitz, 2019).

Emerging data reveal that 14 out of 20 retrogenes derived from 16 X-linked progenitor genes are specifically expressed in testis (Wang, 2004). During meiosis, most X-linked genes become transcriptionally silenced because of meiotic sex chromosome inactivation (MSCI), and their functions can be compensated by corresponding retrogenes (Wang, 2004; Turner, 2015). We previously demonstrated that the X-linked gene *RPL10*, encoding a ribosomal protein, and its autosomal paralog *RPL10L* can functionally compensate for each other *in vitro* and *in vivo* (Jiang et al., 2017). The majority of the retrotransposed genes are inactivated and become pseudogenes, and some of the retrogenes perform important functions during male meiosis (Schrader and Schmitz, 2019). For example, the knockout of the well-known testis-specific retrogenes, such as *Cetn1*, *Cstf2t*, *Pgk2* and *Rpl10l*, can result in spermatogenic failure (Dass et al., 2007; Danshina et al., 2010; Tardif et al., 2010; Avasthi et al., 2013; Jiang et al., 2017).

Generally, ubiquitin-like (UBL) proteins modify their target substrates and may have important functions in spermatogenesis (Schwartz and Hochstrasser, 2003). As a member of the UBL proteins, *Ubl4b* is also an autosomal retrogene with testis-specific expression derived from *Ubl4a*. *Ubl4a* (NM_145405) maps to X chromosome and is ubiquitously expressed in different tissues (Wang et al., 2012), hence regarded as a housekeeping gene. It exerts a significant effect in protein metabolism and the maintenance of cellular homeostasis. For example, UBL4A can maintain the innate immune response through positively regulating NF-κB signaling in dendritic cells and macrophages (Liu et al., 2019). It also plays an antitumor role on autophagy-related proliferation and metastasis in pancreatic ductal adenocarcinoma by directly targeting LAMP1 (Chen et al., 2019a). Intriguingly, *Ubl4a* knockout (KO) mice were viable and during 6 months after the birth, no obvious abnormality was observed in the development and growth (Wang et al., 2012). What’s more, single knockout of *Ubl4a* does not affect spermatogenesis and fertility in mice, suggesting a possible compensatory role by the *Ubl4b* retrogene (Wang et al., 2012). However, the role of *Ubl4b* and its relationship with *Ubl4a* are still needed to be elucidated.

Thus, the current study was focused to investigate *in vivo* function of evolutionarily conserved *Ubl4b* gene by generation of knockout mice. We also obtained double mutant mice that lack both *Ubl4a* and *Ubl4b* to get insights into the association of *Ubl4a* and *Ubl4b in vivo*. Interestingly, we found no alterations in reproductive parameters of *Ubl4b^–/–^* male mice, and *Ubl4a^–/Y^; Ubl4b^–/–^* mice also displayed normal spermatogenesis. Thus, *Ubl4a* and *Ubl4b* are both dispensable for spermatogenesis through the *in vivo* study, which provided a direct evidence for the first time that some X chromosome-derived autosomal retrogenes can be unfunctional in spermatogenesis.

## Materials and Methods

### Phylogenetic Analysis and Selection Analysis of *UBL4A* and *UBL4B*

Entire coding nucleotide and amino acid sequences of *UBL4A* and *UBL4B* in different vertebrates were downloaded from National Center for Biotechnology Information (NCBI). UBL domain-based protein sequences were extracted from UniProt database. The alignments were performed using the online software MultAlin^1^. The phylogenetic construction was performed on MEGA 6.06 software with the following workflow: Muscle was used for the alignment of entire coding nucleotide sequences of *UBL4A* and *UBL4B* in different eukaryotic species with default settings (Edgar, 2004). Phylogenetic trees were constructed by the Neighbor-joining (NJ) method. The parameters used for the tree construction include phylogeny test (bootstrap method with 1,000 replicates), substitutions type (nucleotide), model/method (maximum composite likelihood), substitutions (transitions and transversions), rates among sites (uniform rates), pattern among lineages (same/homogeneous), gaps/missing data treatment (complete deletion), and codon positions (1st, 2nd, 3rd and non-coding sites). To investigate whether *UBL4A* and *UBL4B* genes have undergone statistically significant differences in selection pressures, we employed the branch model in the CodeML program using the phylogenetic analysis by maximum-likelihood (PAML) software version 4.4 with default settings.

### Mouse Models

CRISPR/Cas9 technology was utilized to make *Ubl4b* mutant mice as we described previously (Wang et al., 2013). The following sequence 5′TCAGCACCTACAGGTGCCCG3′ was designed to target the exon 1 of *Ubl4b*. Sanger sequencing of toe biopsies was carried out and heterozygous founder mice were bred to obtain homozygous mice. All mice were nourished with proper food and ddH_2_O, and kept in definite photoperiod (lights on 08:00–20:00). All animal experiments were approved by the Institutional Animal Care Committee of the University of Science and Technology of China. *Primers* that were used for genotyping are listed in Supplementary Table 1.

### Western Blot

Testes from 10-week-old mice were homogenized in lysis buffer (50 mM Tris, pH 7.5, 150 mM NaCl, 0.5% Triton X–100, 5 mM EDTA and 1 mM Na_3_VO_4_) containing protease inhibitors (Roche). Western blot was carried out as described previously (Jiang et al., 2014). The primary antibodies are rabbit anti-β-actin (Abcam, ab8227; 1:3,000), rabbit anti-UBL4A (Yang et al., 2007) and rabbit anti-UBL4B (Yang et al., 2007). The secondary antibody is HRP-conjugated donkey anti-rabbit IgG (BioLegend, 406401; 1:10,000).

### Fertility Test

To check the fertility status, each 10-week-old male mouse was mated with two 8-week-old wild-type (WT) females (C57BL/6J) for 3 months. All the females were monitored for pregnancy. Dates of birth, numbers of pups and offspring sex ratios were recorded for all the litters.

### Hematoxylin and Eosin (H&E) Staining

Adult mice at 10 weeks old were sacrificed, then their testes and epididymides were detached immediately and fixed overnight in Bouin’s solution. Further H&E staining was carried out as explained previously (Jiang et al., 2015). To reduce the experimental variations, all these procedures were performed simultaneously in WT and KO mice.

### Sperm Analysis

For sperm counting, epididymides were removed and chopped into small pieces. After incubation at 37°C for 30 min, a hemocytometer was used for cell counting. For sperm morphology, smear slides were analyzed by H&E staining. The percentages of morphologically normal sperm were quantified with at least 400 sperm examined for each mouse. For sperm motility, cauda epididymides from 10-week-old mice were collected and sperm were incubated in human tubal fluid (HTF) media (Millipore) supplemented with 10% FBS at 37°C for 5 min. Sperm samples were diluted and analyzed using Hamilton Thorne’s Ceros II system.

### Statistical Analysis

Student’s *t*-test was performed to statistically compare the teste/body weight ratios, litter sizes, and sperm parameters. Results were presented as mean ± SEM from at least three mice for each group and *p* < 0.05 was considered significant.

## Results

### Conserved Evolution of *UBL4A* and Its Retrogene *UBL4B*

**FIGURE 1:**
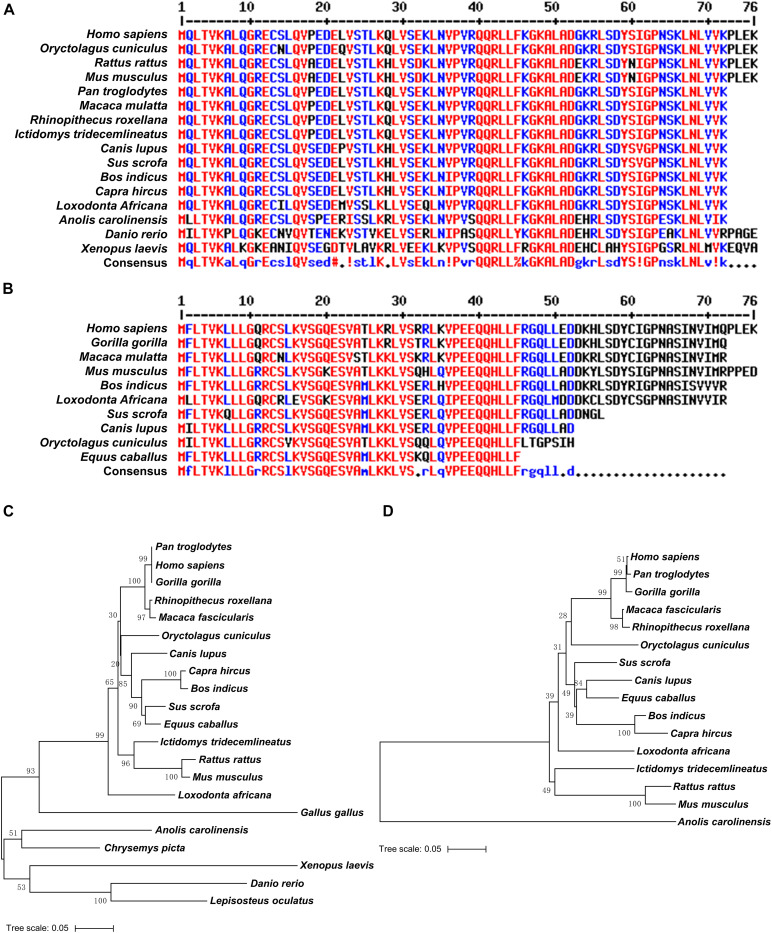
UBL4A and its retrogene UBL4B are evolutionarily conserved. **(A,B)** Amino acid sequence alignments of UBL domains in UBL4A **(A)** and UBL4B **(B)** from various organisms. Residues that are identical appear in red and as uppercase letters in the consensus line. Residues highly similar are indicated by red symbols (!, any one of I and V;%, any one of F and Y; #, any one of N, D, Q, E, B, and Z). Unconserved residues are written in blue or as asterisks in the consensus line. **(C,D)** The phylogenetic trees based on entire coding nucleotide sequences of *UBL4A* **(C)** and *UBL4B* **(D)** in various organisms. The bootstrap confidence values of nodes are displayed.

To explore the origin of *UBL4A* and *UBL4B* individually, the genetic relationships among various organisms were included. Since it has been previously reported that retroposition of *Ubl4b* probably occurred at least 170 million years ago, prior to the radiation of therian mammals (Yang et al., 2007), we collected detailed information (Taxonomy ID, Organism name, Gene ID, Chromosome and Exon count) of *UBL4A* and *UBL4B* among vertebrates from NCBI database (Supplementary Table 2). We found that *UBL4A* genes are detected in vertebrates such as fishes, amphibians, reptiles, birds and mammals, while *UBL4B* members are only present in reptiles and mammals (Supplementary Table 2). Almost the half of the *UBL4A* genes are located on X chromosomes with four exons, while most of the *UBL4B* genes exist on autosomes with only one exon (Supplementary Table 2). The entire coding nucleotide and amino acid sequences of *UBL4A* and *UBL4B* among vertebrates were downloaded from NCBI database. Multiple sequence alignments of the entire coding nucleotide and amino acid sequences revealed a high similarity in *UBL4A* and *UBL4B* (Supplementary Figures 1–4). In particular, amino acid sequence alignments from UBL domains of UBL4A and UBL4B in various organisms showed a higher similarity (Figures 1A,B). Phylogenetic trees were then constructed based on entire coding nucleotide sequences of *UBL4A* and *UBL4B* using MEGA 6.06 software and NJ method, and further revealed that *UBL4A* and *UBL4B* among vertebrates may derive from the corresponding ancestral genes, respectively (Figures 1C,D). Thus, these results based on sequence and phylogenetic analysis indicated that *UBL4A* and *UBL4B* are conserved in vertebrates.

**FIGURE 2:**
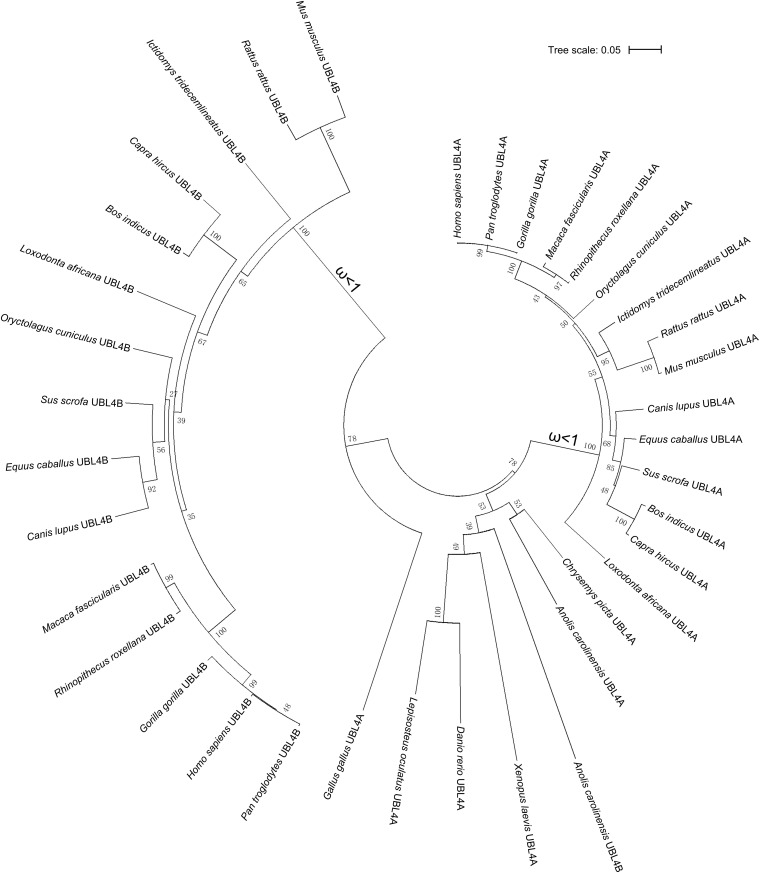
Purifying selection of *UBL4A* and *UBL4B* in various organisms. The phylogenetic tree based on entire coding nucleotide sequences of *UBL4A* and *UBL4B* in various organisms. The number on the branch shows the ω value. The ω values refer to the non-synonymous substitution per non-synonymous site (dN)/synonymous substitution per synonymous site (dS). The bootstrap confidence values of nodes are also displayed.

To trace the evolution of *UBL4A* and *UBL4B*, we performed phylogenetic analysis on *UBL4A* and *UBL4B* among vertebrates simultaneously and found that *UBL4A* and *UBL4B* may originate from a common ancestral gene (Figure 2). Furthermore, *UBL4A* and *UBL4B* in mammalian vertebrates notably gather together, respectively, in the phylogenetic tree (Figure 2). To better understand which selection *UBL4A* and *UBL4B* underwent in the course of evolution, we utilized the branch model in the CodeML program for the phylogenetic analysis with PAML software 4.4. The non-synonymous substitution per non-synonymous site (dN)/synonymous substitution per synonymous site (dS), termed as the ω value, indicates the nature of selective forces. We defined the phylogeny of *UBL4A* in mammalian vertebrates as a foreground branch, the other phylogenies as a background branch, and calculated ω and *p*-values (Figure 2). The ω and *p*-values for above group were 0.07033 and 0.00067, showing that the branch of *UBL4A* in mammalian vertebrates evolves under purifying selection. Similarly, we defined the phylogeny of *UBL4B* in mammalian vertebrates as a foreground branch, the other phylogenies as a background branch, and figured ω and *p*-values (Figure 2). The ω and *p*-values for this group were 0.00303 and 0.00204, revealing that the branch of *UBL4B* in mammalian vertebrates is under strong purifying selection process in evolution. Thus, these results suggested that *UBL4A* and *UBL4B* may have conserved functions in the course of evolution.

### Ubl4b Is Dispensable for Male Fertility and Spermatogenesis

**FIGURE 3:**
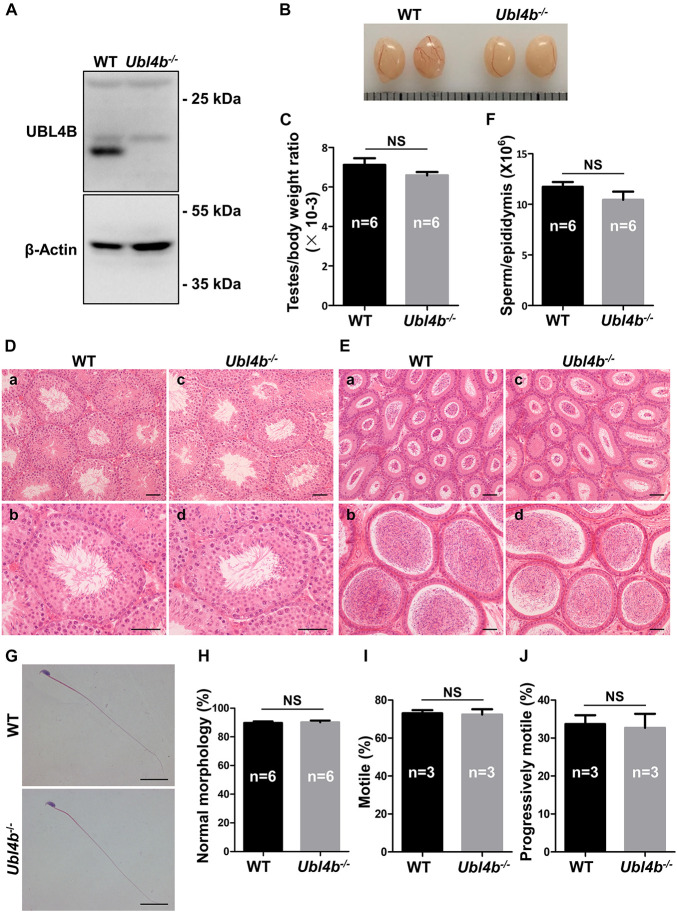
Spermatogenesis in *Ubl4b* knockout mice. **(A)** Western blot analysis of UBL4B expression in testes from 10-week-old WT and *Ubl4b^–/–^* mice. β-Actin was used as the loading control. **(B)** Representative image of testes from 10-week-old WT and *Ubl4b^–/–^* mice. **(C)** Ratios of testes to body weight were determined from 10-week-old WT and *Ubl4b^–/–^* mice. **(D)** H&E staining of testes from 10-week-old WT **(a,b)** and *Ubl4b^–/–^* (c, d) mice. Scale bars, 50 μm. **(E)** H&E staining of caput and cauda epididymides from 10-week-old WT (a, b) and *Ubl4b^–/–^* **(c,d)** mice. Scale bars, 50 μm. **(F)** Average sperm count in unilateral epididymis from 10-week-old WT and *Ubl4b^–/–^* mice. **(G)** H&E staining of sperm in cauda epididymides from 10-week-old WT and *Ubl4b^–/–^* mice. Scale bars, 20 μm. **(H)** Percentages of sperm with normal morphology were shown. **(I,J)** Percentages of motile sperm **(I)** and progressively motile sperm **(J)** from 10-week-old WT and *Ubl4b^–/–^* mice. n, the number of animals. The data shown were represented as the mean ± SEM. Student’s *t*-test was performed between WT and *Ubl4b^–/–^* mice. NS, no significant difference.

In order to investigate the function of *Ubl4b in vivo*, we generated *Ubl4b* knockout mice using CRISPR/Cas9 technology (Supplementary Figure 5A). The knockout of *Ubl4b* was firstly validated by a large deletion at the genomic DNA level (Supplementary Figure 5B). Sanger sequencing confirmed an 80 base pairs (bp) deletion in *Ubl4b*, which introduced a frameshift mutation at amino acid 13 (Supplementary Figure 5C). Then, Western blot confirmed a complete absence of UBL4B protein in *Ubl4b^–/–^* testis, which further confirmed the successful deletion of *Ubl4b* in our gene modified mice (Figure 3A).

*Ubl4b^–/–^* mice were outward normal, and showed normal growth and development similar to their WT littermates. Subsequently, the fertility status was analyzed by keeping WT and *Ubl4b^–/–^* male mice with WT females, respectively. The statistical analysis showed that the *Ubl4b^–/–^* male mice had comparable number of pups per litter and offspring sex ratios to the WT group, which suggested that the deficiency of *Ubl4b* does not affect male fertility (Table 1).

**TABLE 1:** Fertility assay.

Genotype	Mating period (months)	No. of fertile males/No. of mice tested	Average pups/litter	Offspring sex ratio (% males)
WT	3	3/3	7.80 ± 0.32	0.49 ± 0.12
*Ubl4b^–/–^*	3	3/3	8.05 ± 0.46^NS^	0.50 ± 0.08^NS^

**Each male was engaged with two WT females. Student’s t-test was used for comparison of average pups/litter and offspring sex ratios (% males) between WT and Ubl4b^–/–^ mouse groups. NS, no significant difference. Data were presented as mean ± SEM.**

To determine the possible role of *Ubl4b* in spermatogenesis, we firstly analyzed the testes from adult WT and *Ubl4b^–/–^* mice and found no any noteworthy between-group difference in the testis morphology and testes to body weight ratios (Figures 3B,C). Moreover, the histology of testes by H&E staining revealed intact seminiferous tubule architecture along with the presence of all types of germ cells from spermatogonia to spermatozoa in *Ubl4b^–/–^* mice (Figure 3D). Additionally, the cauda epididymides of adult *Ubl4b^–/–^* mice were filled with abundant mature spermatozoa (Figure 3E). Unilateral epididymal sperm from adult WT and *Ubl4b^–/–^* mice were counted and statistical analysis showed that there is no significant difference (Figure 3F). Then we further compared the sperm parameters of WT and *Ubl4b^–/–^* mice. Epididymal sperm from adult *Ubl4b^–/–^* mice displayed normal morphology, similar to that of WT mice (Figures 3G,H). Furthermore, we did not observe any remarkable difference in sperm motility between adult WT and *Ubl4b^–/–^* mice (Figures 3I,J). Taken altogether, these findings suggested that *Ubl4b* is not required for normal fertility and spermatogenesis in mice.

### Double Knockout of *Ubl4a* and *Ubl4b* Has No Effect on Fertility and Spermatogenesis

**FIGURE 4:**
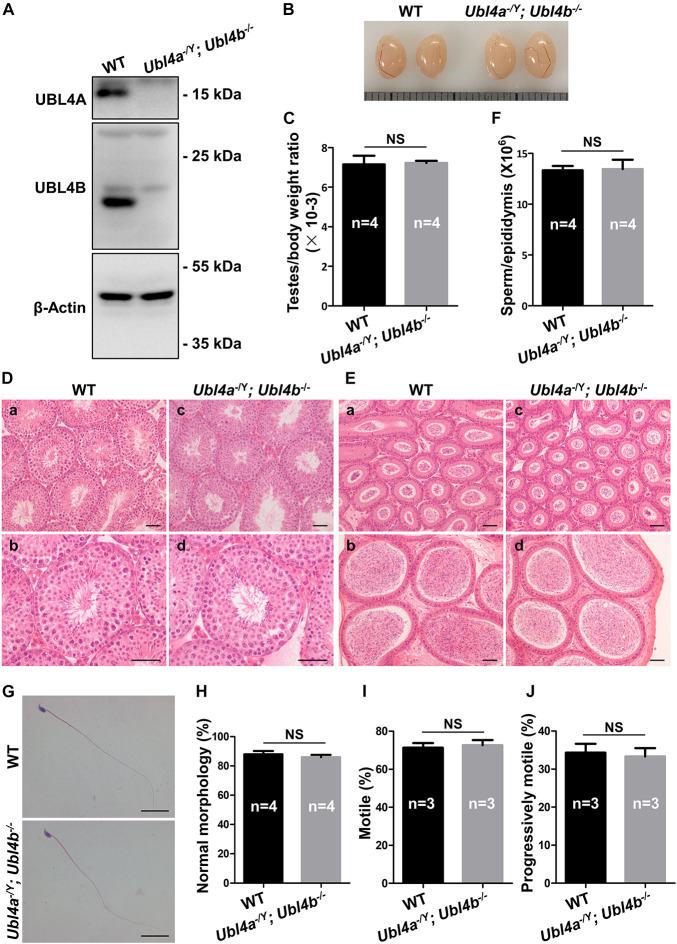
Spermatogenesis in *Ubl4a* and *Ubl4b* double knockout mice. **(A)** Western blot analysis of UBL4A and UBL4B expression in testes from 10-week-old WT and *Ubl4a^–/Y^; Ubl4b^–/–^* mice. β-Actin was used as the loading control. **(B)** Representative image of testes from 10-week-old WT and *Ubl4a^–/Y^; Ubl4b^–/–^* mice. **(C)** Ratios of testes to body weight were determined from 10-week-old WT and *Ubl4a^–/Y^; Ubl4b^–/–^* mice. **(D)** H&E staining of testes from 10-week-old WT **(a,b)** and *Ubl4a^–/Y^; Ubl4b^–/–^* **(c,d)** mice. Scale bars, 50 μm. **(E)** H&E staining of caput and cauda epididymides from 10-week-old WT **(a,b)** and *Ubl4a^–/Y^; Ubl4b^–/–^* **(c,d)** mice. Scale bars, 50 μm. **(F)** Average sperm count in unilateral epididymis from 10-week-old WT and *Ubl4a^–/Y^; Ubl4b^–/–^* mice. **(G)** H&E staining of sperm in cauda epididymides from 10-week-old WT and *Ubl4a^–/Y^; Ubl4b^–/–^* mice. Scale bars, 20 μm. **(H)** Percentages of sperm with normal morphology were shown. **(I,J)** Percentages of motile sperm **(I)** and progressively motile sperm **(J)** from 10-week-old WT and *Ubl4a^–/Y^; Ubl4b^–/–^* mice. n, the number of animals. The data shown were represented as the mean ± SEM. Student’s *t*-test was performed between WT and *Ubl4a^–/Y^; Ubl4b^–/–^* mice. NS, no significant difference.

As *Ubl4b^–/–^* mice showed normal fertility and spermatogenesis, we hypothesized that *Ubl4a* and *Ubl4b* may be functionally redundant. In order to investigate the functions of these two genes *in vivo*, we crossed *Ubl4a^–/Y^* mice with *Ubl4b^–/–^* female mice (Wang et al., 2012). After two generations of breeding, we obtained desired *Ubl4a^–/Y^; Ubl4b^–/–^* mice. The genotypes of *Ubl4a^–/Y^; Ubl4b^–/–^* mice were firstly verified on genomic level (Supplementary Figure 6). Then, the observation of complete absence of both UBL4A and UBL4B proteins in *Ubl4a^–/Y^; Ubl4b^–/–^* testes confirmed the successful deletion of *Ubl4a* and *Ubl4b* simultaneously (Figure 4A).

After the confirmation of successful generation of *Ubl4a^–/Y^; Ubl4b^–/–^* mice, we assessed the fertility of WT and dKO males. Mice lacking both *Ubl4a* and *Ubl4b* showed normal growth and development. The average pups per litter and offspring sex ratios of adult *Ubl4a^–/Y^; Ubl4b^–/–^* mice were not significantly different from those in WT group (Table 2).

**TABLE 2:** Fertility assay.

Genotype	Mating period (months)	No. of fertile males/No. of mice tested	Average pups/litter	Offspring sex ratio (% males)
WT	3	3/3	7.60 ± 0.23	0.52 ± 0.09
*Ubl4a^–/Y^; Ubl4b^–/–^*	3	3/3	7.91 ± 0.56^NS^	0.56 ± 0.08^NS^

**Each male was engaged with two WT females. Student’s t-test was used for comparison of average pups/litter and offspring sex ratios (% males) between WT and Ubl4a^–/Y^; Ubl4b^–/–^ mouse groups. NS, no significant difference. Data were presented as mean ± SEM.**

The testis morphology and testes to body weight ratios of adult *Ubl4a^–/Y^; Ubl4b^–/–^* mice did not exhibit notable difference from those of WT group (Figures 4B,C). Subsequently, histological analysis showed normal spermatogenesis in seminiferous tubules and abundant sperm in epididymides of *Ubl4a^–/Y^; Ubl4b^–/–^* mice (Figures 4D,E). Epididymal sperm count did not significantly differ between adult WT and *Ubl4a^–/Y^; Ubl4b^–/–^* mice (Figure 4F). Moreover, sperm morphology was analyzed by H&E staining and we found no obvious difference between adult WT and *Ubl4a^–/Y^; Ubl4b^–/–^* mice (Figures 4G,H). Additionally, sperm motility of adult WT and *Ubl4a^–/Y^; Ubl4b^–/–^* mice was indistinguishable from each other (Figures 4I,J). Altogether, these data strongly demonstrated that the simultaneously deletion of *Ubl4a* and its retrogene, *Ubl4b*, did not affect male fertility and spermatogenesis in mice.

## Discussion

The mammalian genome contains various retrotransposed genes and most of them are transcriptionally silent (Venter et al., 2001; Brosius, 2019). Here we showed that *Ubl4a* and *Ubl4b* are subject to purifying selection in phylogenetic analysis and may have conserved functions in evolution. In order to determine the functional role of testis-specific X-derived retrogene *Ubl4b* in mice, we generated a knockout mouse line by CRISPR/Cas9 technology and found no alternations in reproductive parameters. Subsequently, to explore the possibility of compensation by the ubiquitously expressed *Ubl4a* gene, we further produced double mutant mice lacking both *Ubl4a* and *Ubl4b* genes. Interestingly, adult *Ubl4a^–/Y^; Ubl4b^–/–^* mice also displayed normal fertility and spermatogenesis, showing dispensable roles of both genes in mouse reproduction.

During male meiosis, most of the X-linked genes are transcriptionally silent due to formation of heterochromatin XY body and the functions of these genes can be compensated by their corresponding autosomal retrogenes so that spermatocytes can complete meiosis (Richler et al., 1994; Turner et al., 2002). The expression profile indicates that *Ubl4a* transcription is transiently silenced during MSCI, while *Ubl4b* mRNA could be detected from pachytene stage, indicating that *Ubl4b* may compensate the silence of *Ubl4a* in MSCI (Yang et al., 2007). Previously knockout of such types of retrogenes, including *Cetn1*, *Cstf2t*, *Pgk2* and *Rpl10l*, always resulted in spermatogenic abnormalities and male infertility (Dass et al., 2007; Danshina et al., 2010; Tardif et al., 2010; Avasthi et al., 2013; Jiang et al., 2017). However, the deletion of either *Ubl4a* or *Ubl4b* in mice showed no overt abnormalities in spermatogenesis and fertility (Wang et al., 2012). What is more, the mice with dKO of *Ubl4a* and *Ubl4b* also displayed normal spermatogenesis with no obvious alternation in fertility status. Thus, our unexpected results revealed that both genes have no roles in fertility and spermatogenesis.

Several hypotheses have been described about the evolution and function of retrogenes in mammals. One of them is the compensation hypothesis of X-derived retrogenes, for example, we previously demonstrated that the X-linked gene *RPL10*, encoding a ribosomal protein, and its autosomal paralog *RPL10L* can functionally compensate for each other (Jiang et al., 2017). Another hypothesis about retrogene evolution is the specialization hypothesis in which many X-originated retrogenes have evolved novel functions to meet the special needs of germ cells. This is supported by different mechanisms including alternative splicing, polyadenylation and apoptosis in somatic and germ cells, and the obvious examples of genes are *hnRNPG-T*, *CSTF2T*, and *BIRC8* (Elliott et al., 2000; Richter et al., 2001; Dass et al., 2002; Grozdanov et al., 2018; Bousoik et al., 2019; Zhou et al., 2019; Yan et al., 2021). RBMX protein has been implicated in splice site selection, and RBMX-derived retrogene product, hnRNPG-T, could be specialized in alternative splicing in germ cells (Elliott et al., 2000; Zhou et al., 2019; Yan et al., 2021). CSTF2T could function in the non-canonical polyadenylation of mRNAs in germ cells (Dass et al., 2002; Grozdanov et al., 2018). Another well-known hypothesis regarding X-derived retrogenes is the haploid syncytium hypothesis based on non-equality of sex chromosomes in haploid germ cells and this disequilibrium is fulfilled by the presence of “back up” autosomal retrogenes (Dahl et al., 1990; Casola and Betran, 2017). An explanatory example of this hypothesis is that *G6pd2*, a spermatid specific expressed gene, is considered as a “back up” gene for equal amount of its product for both X and Y bearing spermatids (Hendriksen et al., 1997; Homolka et al., 2011). In addition, the possession of novel adjacent sequences in the form of untranslated regions (UTRs) to attain a special regulatory mechanism was put forward to explain the emergence of retrogenes from X chromosomes (Shiao et al., 2007). The function and evolution of these autosomal retrogenes derived from X-linked genes might be unrelated with MSCI during male meiosis and an explanatory example is that *CHML*, an ubiquitous expressed gene, has been reported to have function in cancer but not in reproduction (Chen et al., 2019b). However, none of these hypotheses can explain the observation that both X-linked *Ubl4a* and its autosomal retrogene, *Ubl4b* are dispensable in spermatogenesis.

In the current study, we found *Ubl4a* and *Ubl4b* double knockout mice evinced normal fertility and spermatogenesis similar to *Ubl4a* and *Ubl4b* single knockout mice, which does not support the existing hypotheses for X-derived retrogenes. We deduce that the evolutionarily conserved *Ubl4a* and *Ubl4b* genes may initially have essential functions in spermatogenesis, but are no longer required during evolution. On the other hand, it is also plausible that *Ubl4b* may be derived from insertion of *Ubl4a* mRNA into autosomal genomes by accident, which did not lead to any deleteriousness and thus was kept during evolution or has not yet begun its degeneration to oblivion. What is more, *Ubl4b* and *Ubl4b* may be hitchhiked by other genes under purifying selection in the genome. For example, *Ubl4a* is close to mouse *Slc10a3*, while *Ubl4b* is near *Slc6a17*, in which both *Slc10a3* and *Slc6a17* are regarded as housekeeping genes that play important functions in many biological processes. Alternatively, a few substitutions are sufficient to acquire a new function, and UBL4A and UBL4B may have obtained some important functions that is not related to male fertility. Thus, we propose a new “junk” hypothesis that some autosomal retrogenes derived from X-linked progenitor genes may not have functions in spermatogenesis, which is supported by the *in vivo* study of *Ubl4a* and *Ubl4b*. In future, more intensive and sophisticated studies are needed to reinforce this hypothesis by other groups of X-linked genes and autosomal counterparts. Additionally, all the examinations were performed under normal laboratory mating conditions and challenging the UBL system in mice, such as a toxicology study, deserves further consideration.

## Conclusion

We generated *Ubl4b^–/–^* and *Ubl4a^–/Y^; Ubl4b^–/–^* mice which displayed normal fertility and spermatogenesis, proposing that both X-linked *Ubl4a* and its autosomal counterpart *Ubl4b* are not necessary for male fertility and providing the first direct evidence for the “junk” hypothesis regarding X-derived retrogenes.

## Data Availability Statement

The original contributions presented in the study are included in the article/Supplementary Material, further inquiries can be directed to the corresponding author/s.

## Ethics Statement

The animal study was reviewed and approved by the Institutional Animal Care Committee of the University of Science and Technology of China.

## Author Contributions

QS, XJ, and CY: conceptualization. XJ and CY: formal analysis. CY and RD: investigation. QS, ZC, and CD: resources. RK and CY: writing—original draft preparation. XJ, HM, and CY: writing—review and editing. QS: project administration and funding acquisition. All authors contributed to the article and approved the submitted version.

## Conflict of Interest

The authors declare that the research was conducted in the absence of any commercial or financial relationships that could be construed as a potential conflict of interest.

## Footnotes

1 http://multalin.toulouse.inra.fr/multalin/multalin.html

## Supplementary Material

Supplementary Figure 1 CDS sequence alignment of *UBL4A* in various organisms.

Supplementary Figure 2 CDS sequence alignment of *UBL4B* in various organisms.

Supplementary Figure 3 Amino acid sequence alignment of UBL4A in various organisms. Residues that are identical appear in red and as uppercase letters in the consensus line. Residues highly similar are indicated by red symbols (!, any one of I and V; $, any one of L and M;%, any one of F and Y; #, any one of N, D, Q, E, B, and Z). Unconserved residues are written in blue or as asterisks in the consensus line.

Supplementary Figure 4 Amino acid sequence alignment of UBL4B in various organisms. Residues that are identical appear in red and as uppercase letters in the consensus line. Residues highly similar are indicated by red symbols (!, any one of I and V; $, any one of L and M; #, any one of N, D, Q, E, B, and Z). Unconserved residues are written in blue or as asterisks in the consensus line.

Supplementary Figure 5 Generation of *Ubl4b* knockout mice. **(A)** Schematic strategy of sgRNA designed for *Ubl4b* knockout. Diagram of the mouse *Ubl4b* locus is based on Ensembl data (transcript ENSMUST00000052853.7), with blue and white bars representing coding and non-coding regions, respectively. PAM, protospacer adjacent motif. **(B)** PCR using genomic DNA (gDNA) from WT and *Ubl4b^–/–^* mice was performed to verify the deletion of *Ubl4b* in the knockout mice. M, marker. **(C)** Representative Sanger sequencing chromatograms confirmed the genotypes of WT and *Ubl4b^–/–^* mice. Red arrow above the chromatograms in aligned sequences indicates the deletion.

Supplementary Figure 6 Generation of *Ubl4a* and *Ubl4b* double knockout mice. PCR using gDNA from WT and *Ubl4a^–/Y^; Ubl4b^–/–^* mice was performed to verify the deletions of *Ubl4a* and *Ubl4b* in the double knockout mice. M, marker.
