# Supplementary material for: The Dispensable Roles of X-Linked Ubl4a and Its Autosomal Counterpart Ubl4b in Spermatogenesis Represent a New Evolutionary Type of X-Derived Retrogenes
Source: Front Genet. 2021 Jun 25;12:689902. doi: 10.3389/fgene.2021.689902 (PMC8267814; doi:10.3389/fgene.2021.689902)
Supplement: Supplementary file 1 [file Table_1.DOCX]

| **Table S1. Primers used for mouse genotyping.** | | |
| --- | --- | --- |
| **Primers** | **Sequence** | **Product size (bp)** |
| *Ubl4b*-Check-FW | TACACCTTCAGGGCAACTCC | WT: 416  KO : 336 |
| *Ubl4b*-Check-RV | AGACGCTCCTCATGCTCTTG |  |
| *Ubl4a*-Check-FW | GACCGTTGGTGTTTGCGTTG | WT: 523  KO : 300 |
| *Ubl4a*-Check-RV | GAGTGAAGCACTGCAGCATCTG |  |
