# Supplementary material for: The Dispensable Roles of X-Linked Ubl4a and Its Autosomal Counterpart Ubl4b in Spermatogenesis Represent a New Evolutionary Type of X-Derived Retrogenes
Source: Front Genet. 2021 Jun 25;12:689902. doi: 10.3389/fgene.2021.689902 (PMC8267814; doi:10.3389/fgene.2021.689902)
Supplement: Supplementary file 2 [file Table_2.DOCX]

**Table S2. *UBL4A* orthologs and their corresponding *UBL4B* paralogs.**

| List number | Taxonomy ID | Organism name | *UBL4A* | | | *UBL4B* | | |
| --- | --- | --- | --- | --- | --- | --- | --- | --- |
|  |  |  | Gene ID | Chromosome | Exon count | Gene ID | Chromosome | Exon count |
| 1 | 9606 | *Homo sapiens* | 8266 | X | 4 | 164153 | 1 | 1 |
| 2 | 9598 | [*Pan troglodytes*](https://www.ncbi.nlm.nih.gov/Taxonomy/Browser/wwwtax.cgi?id=9598) | 473855 | X | 4 | 457124 | 1 | 1 |
| 3 | 9595 | [*Gorilla gorilla*](https://blast.ncbi.nlm.nih.gov/Blast.cgi#alnHdr_1099312248) | 101144868 | X | 4 | 101136176 | 1 | 1 |
| 4 | 9544 | [*Macaca mulatta*](https://blast.ncbi.nlm.nih.gov/Blast.cgi#alnHdr_388454554) | 700643 | X | 4 | 701055 | 1 | 1 |
| 5 | 61621 | *Rhinopithecus bieti* | 108518778 | Unknown | 4 | 108528059 | Unknown | 1 |
| 6 | 10116 | *Rattus norvegicus* | 293864 | X | 4 | 103691676 | 2 | 1 |
| 7 | 10090 | [*Mus musculus*](https://www.ncbi.nlm.nih.gov/Taxonomy/Browser/wwwtax.cgi?mode=Info&id=10090&lvl=3&lin=f&keep=1&srchmode=1&unlock) | 27643 | X | 4 | 67591 | 3 | 1 |
| 8 | 43179 | *Ictidomys tridecemlineatus* | 101978264 | X | 4 | 101970784 | Unknown | 1 |
| 9 | 9986 | *Oryctolagus cuniculus* | 100328764 | Unknown | 4 | 100338070 | 13 | 1 |
| 10 | 9615 | *Canis lupus familiaris* | 612563 | X | 4 | 100856125 | 6 | 1 |
| 11 | 9796 | *Equus caballus* | 100059665 | X | 4 | 100062023 | 5 | 1 |
| 12 | 9785 | *Loxodonta africana* | 100662061 | Unknown | 4 | 100667780 | Unknown | 1 |
| 13 | 9925 | *Capra hircus* | 102173378 | Unknown | 5 | 102184490 | 3 | 1 |
| 14 | 9031 | *Gallus gallus* | 107050156 | Unknown | Unknown | - | - | - |
| 15 | 28377 | *Anolis carolinensis* | 100563261 | 1 | 4 | 100554680 | 4 | 1 |
| 16 | 8479 | *Chrysemys picta* | 101939297 | Unknown | 4 | - | - | - |
| 17 | 8364 | *Xenopus tropicalis* | 100125140 | 8 | 4 | - | - | - |
| 18 | 7955 | *Danio rerio* | 393270 | 8 | 4 | - | - | - |
| 19 | 7918 | *Lepisosteus oculatus* | 102697858 | LG1 | 4 | - | - | - |

LG, Linkage group.
