# Supplementary figures and images for: The Dispensable Roles of X-Linked Ubl4a and Its Autosomal Counterpart Ubl4b in Spermatogenesis Represent a New Evolutionary Type of X-Derived Retrogenes
Source: Front Genet. 2021 Jun 25;12:689902. doi: 10.3389/fgene.2021.689902 (PMC8267814; doi:10.3389/fgene.2021.689902)

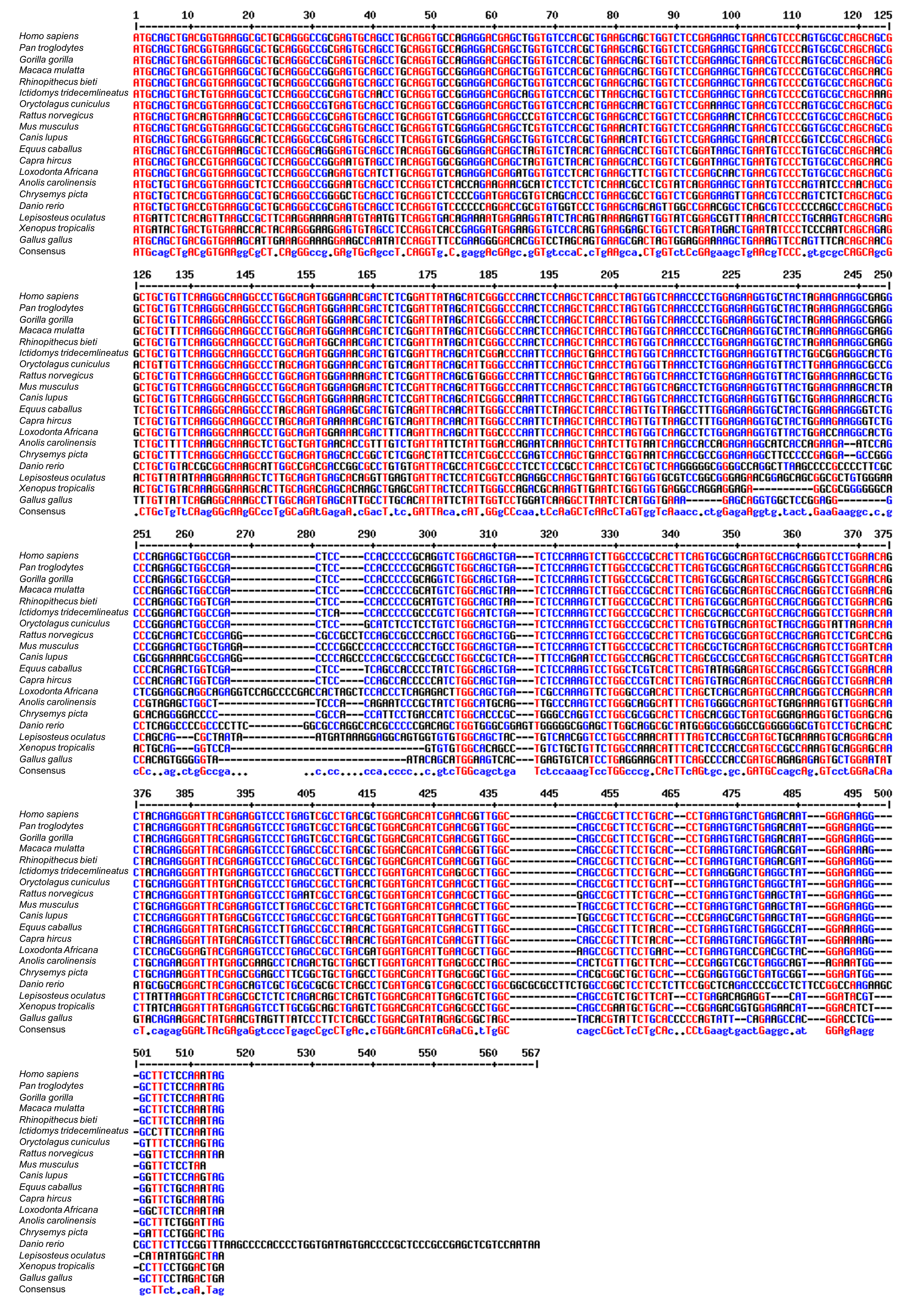

Supplement: Supplementary Figure 1 — CDS sequence alignment of UBL4A in various organisms. [file Image_1.TIF]

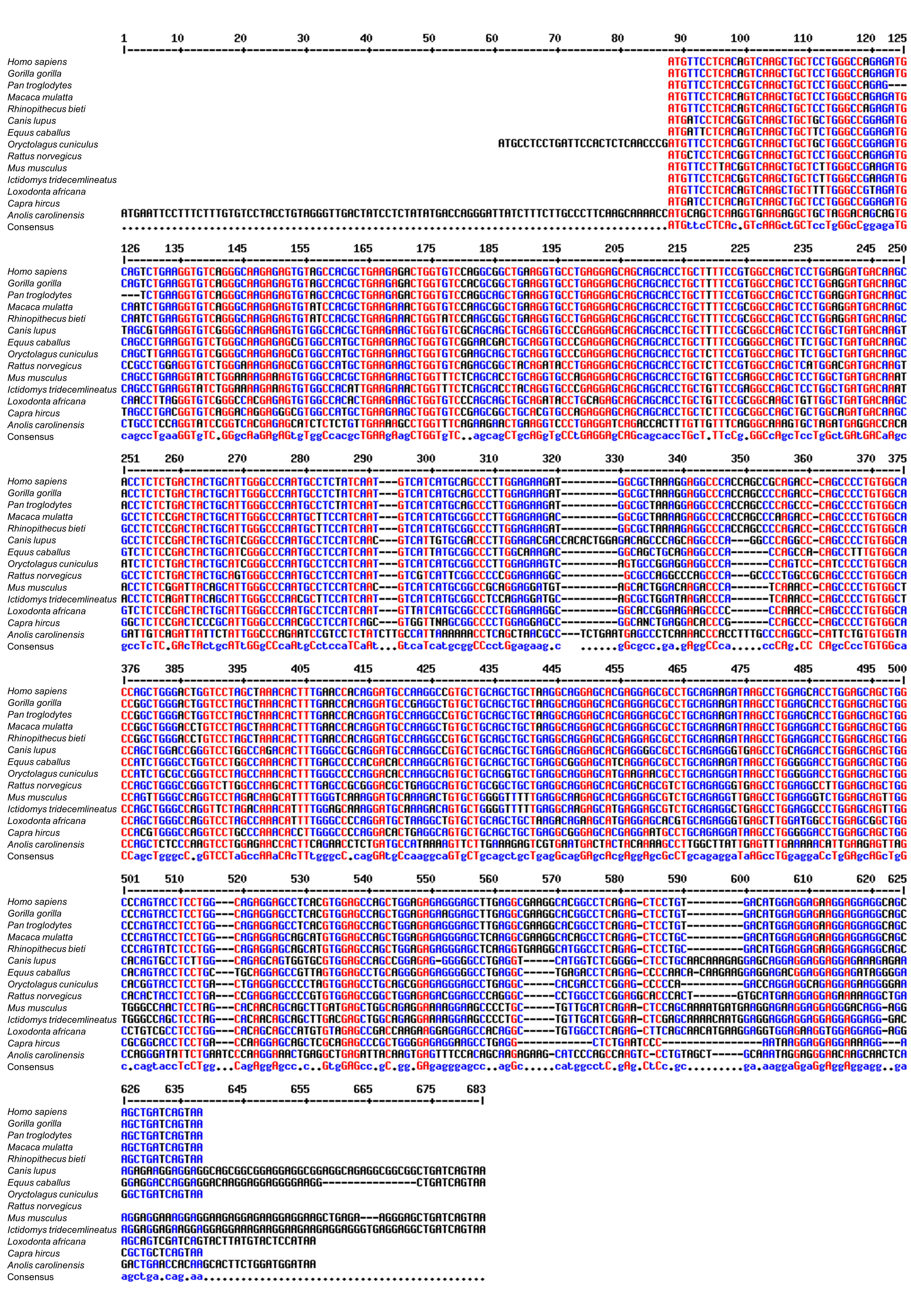

Supplement: Supplementary Figure 2 — CDS sequence alignment of UBL4B in various organisms. [file Image_2.TIF]

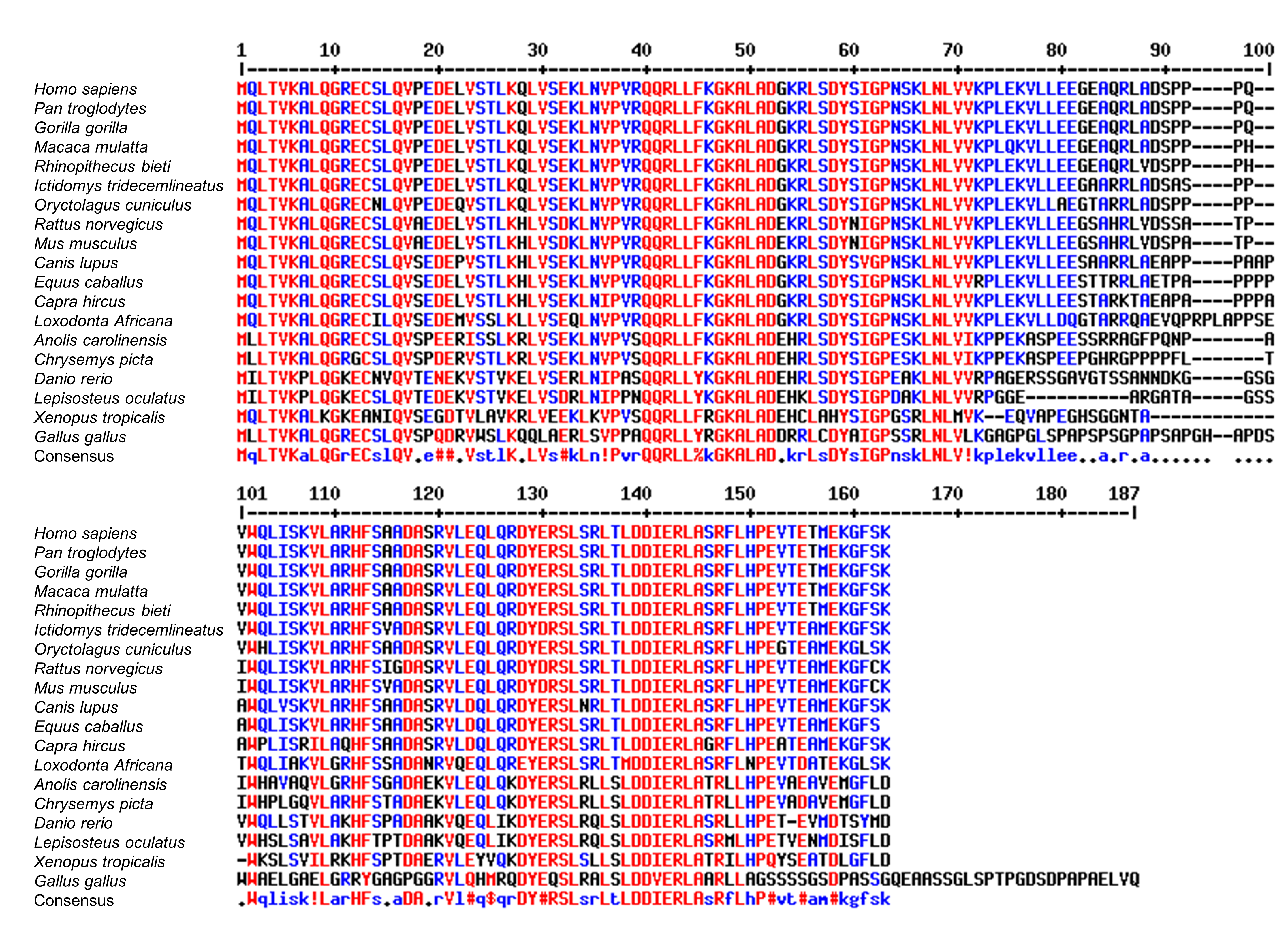

Supplement: Supplementary Figure 3 — Amino acid sequence alignment of UBL4A in various organisms. Residues that are identical appear in red and as uppercase letters in the consensus line. Residues highly similar are indicated by red symbols (!, any one of I and V; $, any one of L and M;%, any one of F and Y; #, any one of N, D, Q, E, B, and Z). Unconserved residues are written in blue or as asterisks in the consensus line. [file Image_3.TIF]

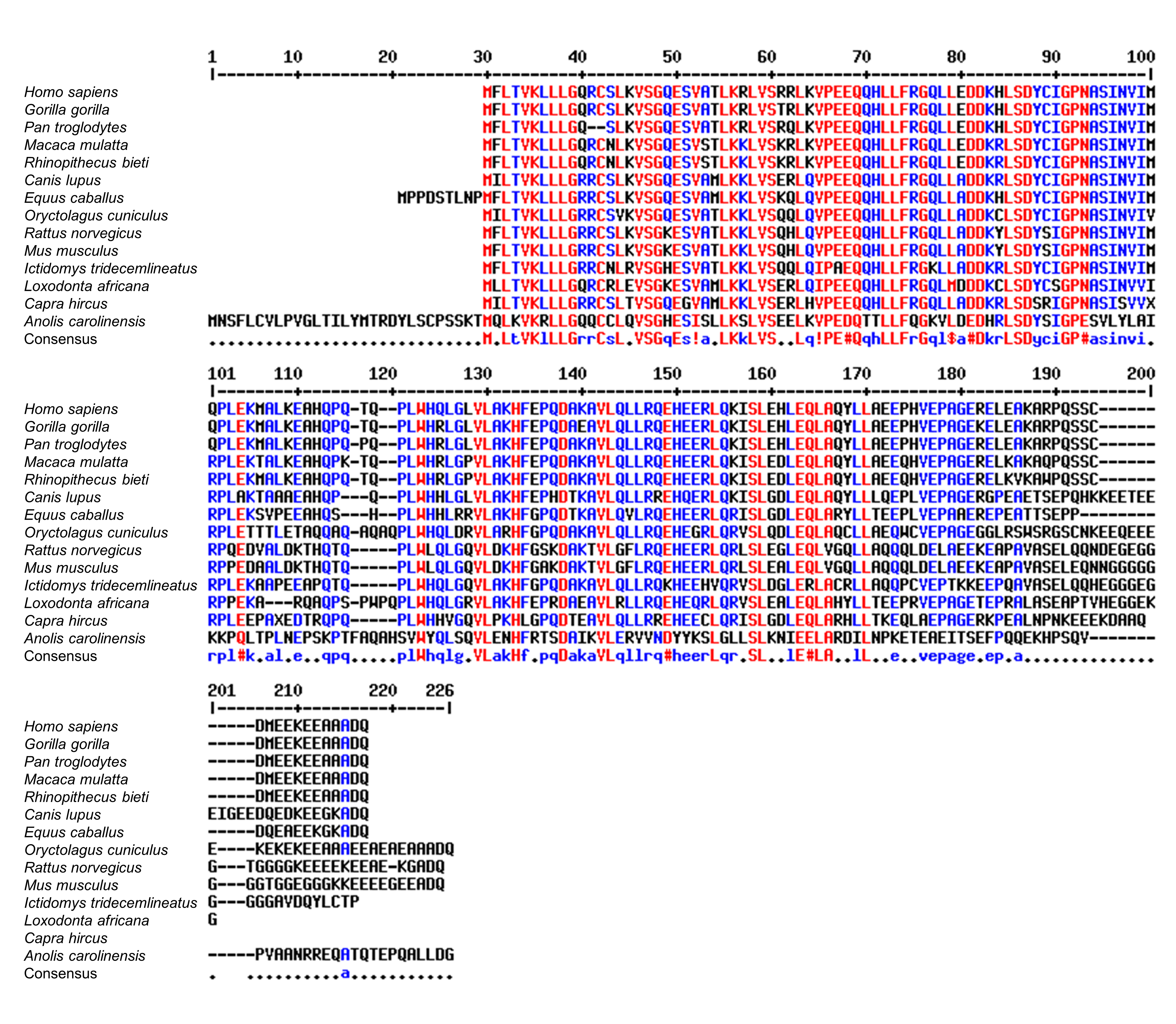

Supplement: Supplementary Figure 4 — Amino acid sequence alignment of UBL4B in various organisms. Residues that are identical appear in red and as uppercase letters in the consensus line. Residues highly similar are indicated by red symbols (!, any one of I and V; $, any one of L and M; #, any one of N, D, Q, E, B, and Z). Unconserved residues are written in blue or as asterisks in the consensus line. [file Image_4.TIF]

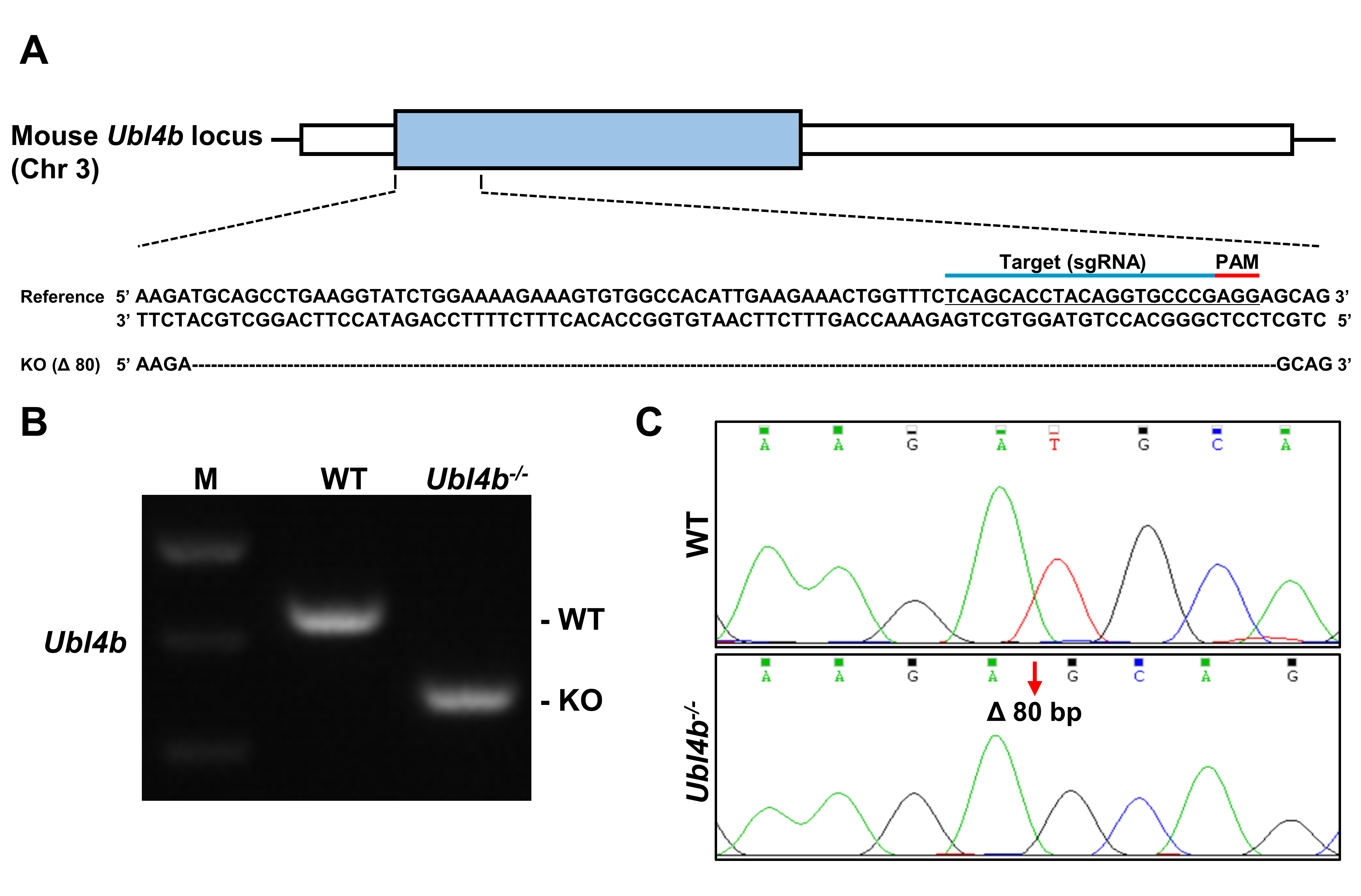

Supplement: Supplementary Figure 5 — Generation of Ubl4b knockout mice. (A) Schematic strategy of sgRNA designed for Ubl4b knockout. Diagram of the mouse Ubl4b locus is based on Ensembl data (transcript ENSMUST00000052853.7), with blue and white bars representing coding and non-coding regions, respectively. PAM, protospacer adjacent motif. (B) PCR using genomic DNA (gDNA) from WT and Ubl4b–/– mice was performed to verify the deletion of Ubl4b in the knockout mice. M, marker. (C) Representative Sanger sequencing chromatograms confirmed the genotypes of WT and Ubl4b–/– mice. Red arrow above the chromatograms in aligned sequences indicates the deletion. [file Image_5.TIF]

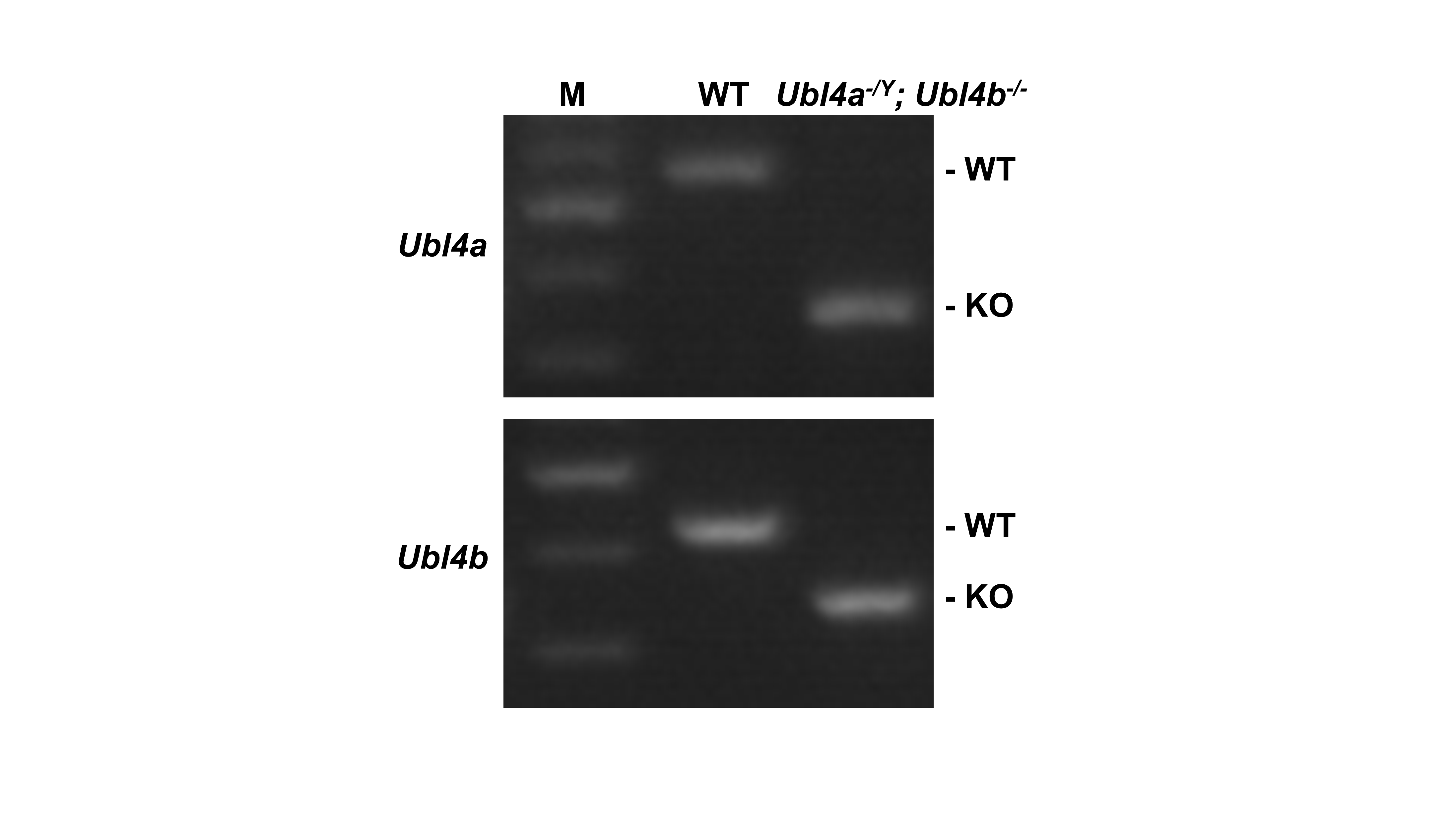

Supplement: Supplementary Figure 6 — Generation of Ubl4a and Ubl4b double knockout mice. PCR using gDNA from WT and Ubl4a–/Y; Ubl4b–/– mice was performed to verify the deletions of Ubl4a and Ubl4b in the double knockout mice. M, marker. [file Image_6.TIF]
